# Test study and molecular dynamics simulation of Fe^3+^ modified TiO_2_ absorbing automobile exhaust

**DOI:** 10.1371/journal.pone.0263040

**Published:** 2022-01-26

**Authors:** Feng Lai, Hongliang Zhang, Kongfa Zhu, Man Huang

**Affiliations:** 1 Key Laboratory for Special Area Highway Engineering of Ministry of Education, Chang’an University, Xi’an, Shaanxi, PR China; 2 Guangdong Nanyue Transportation Huai Yang Expressway, Guangzhou, Guangdong, PR China; Mohanlal Sukhadia University, INDIA

## Abstract

With the growth of the economy, the number of automobiles on the road is fast growing, resulting in substantial environmental pollution from exhaust gas emissions. In the automobile factory, some improvements have been achieved by constructing devices to degrade automobile exhaust. However, although most of the vehicle exhaust emissions have met the national standards, the exhaust gas is superimposed at the same time period due to the increasing traffic volume, making the exhaust emissions seriously reduce the air quality. Therefore, the scholars in the road field began to study new road materials to degrade vehicle exhaust, which has gradually become one of the effective ways to reduce automobile exhaust. Photocatalyst materials have been widely concerned because of their ability to oxidize harmful gases by solar photocatalysis. Yet, the effect has been not satisfactory because of the small light response range of photocatalyst material, which restricts the catalytic effect. In this study, this paper attempts to use Fe^3+^ to modify the TiO_2_, which is one of the main photocatalytic materials, to expand the range of light reaction band and to improve the degradation effect of automobile exhaust. The degradation effects of ordinary TiO_2_ and modified TiO_2_ on automobile exhaust were compared by test system in the laboratory. The results show that the modified TiO_2_ can effectively improve the performance of vehicle exhaust degradation. Moreover, the molecular dynamics method was used to establish the channel model of TiO_2_, and the dynamic process of automobile exhaust diffusion and absorption was simulated. The diffusion law and adsorption process of different types of automobile exhaust gas such as NO, CO, and CO_2_ in the TiO_2_ channel were analyzed from the molecular scale through the radial concentration distribution and adsorption energy.

## 1. Introduction

With the improvement of the economic level and the rapid development of automobile manufacturing industry, the number of automobiles has increased rapidly, resulting in substantial environmental pollution from automobile emissions [1]. Also, the automobile exhaust is the one of the main cause of weather disaster smog. Smog is mainly composed of carbon monoxide, sulphur and nitrogen oxides, which can be produced by incomplete burned fuel with the car moving [2]. Hong, Y. found that automobile exhaust contributed significantly to air pollution and haze, accounting for about 38% [3]. Similarly, Wang, F.C., using Fault Tree Analysis (FTA), indicated that the vehicle exhaust emissions had the great impacts on urban smog, with the contribution ratio reaching 38.4% [4]. Air pollution could cause some harm to respiratory and immune functions of the human body, reducing lung function and increasing the incidence rate of chronic bronchitis and asthma. Automobile exhaust mainly contains carbon monoxide (CO), hydrocarbons (HC), nitrogen oxides (NO, NO_2_) and particulate pollutants. In many air quality monitoring centers in big cities, automobile exhaust has become the primary air pollutant.

How to lessen the environmental pollution caused by automotive emissions has become a hot topic among scholars. In addition to build vehicle devices, improving road materials to reduce the impact of exhaust on the environment has become a new direction. At present, nano photocatalytic materials have been used as new materials for air purification due to their good photocatalytic activity, chemical stability and recyclability. The main photocatalyst materials are semiconductor oxides (ZnO, TiO_2_, SnO_2_, ZrO_2_) and semiconductor sulfides (CdS, PdS). Among them, TiO_2_ is widely used because it has many advantages, such as fast degradation rate, mild reaction conditions, low price, non-toxic [5,6]. However, the bandgap of TiO_2_ is about 3.2eV and an corresponding excitation wavelength is 387nm, and it belongs to the ultraviolet region, which only make up less than 5% of the solar spectrum. Therefore, it is necessary to modify it to expand the range of light reaction band and to improve the catalytic effect.

There are many ways to modify the TiO_2_, such as metal ion doping, noble metal deposition, composite with other semiconductors, modification in its thin film by post treatment, etc. Among them, the noble metal deposition method is to deposit Ru, Pt, Au on the surface of TiO_2_. This modified way can obviously improve the degradation rate of some organic compounds, but the cost is relatively high. The semiconductor composite method is to grind and dissolve the semiconductor with a narrow bandgap and high conduction band position and disperse it in a continuous matrix with wide bandgap photocatalyst materials. Through recombination, the separation and expansion of charge can be promoted. However, it is necessary to adjust the ratio of the two materials in order to obtain the optimal bandgap, which increases the difficulty of the method and is not conducive to its popularization and application. The properties of titanium dioxide films, such as optical and electrical properties, can be tailored by post treatment in various thermal atmospheric conditions, primarily including thermal annealing in air and vacuum, to improve the catalytic efficiency, and the treated film is widely applied as optical window in Cd-based and electron transport layer in perovskite solar cells [7–10]. Although its modification effect is superior, its procession is complicated and requirements on equipment is complex. The method of doping metal ions can cause lattice distortion, form defects, affect the motion of electron-hole pairs, change the band structure of TiO_2_, and finally change the photocatalytic activity of TiO_2_. In previous studies, this method is widely used because of its simplicity and economy.

El-Bahy, Z.M., et al compared the effect of TiO_2_ doped with lanthanide ion (La^3+^, Nd^3+^, Sm^3+^, Eu^3+^, Gd^3+^, and Yb^3+^) on photodegradation and pointed out that lanthanide ions can improve the photocatalytic effect of TiO_2_, especially Gd^3+^ [11]. Doping TiO_2_ with Fe^3+^, Mo^5+^, Ru^3+^, Os^3+^, Re^5+^, V^4+^and Rh^3+^ also improved the photocatalytic effect, in contrast, Co^3+^ and Al^3+^ had opposite effect, which decreased the photoactivity [12]. Further, Wang, X.C., et al. studied the promoting effect of multi‑transition metals (Mo, Ce, Cu, Fe, W, Zr, P) doped with TiO_2_, indicating the catalytic effects of transition metals doped with TiO_2_ following the order of Mo > Fe≈Zr > Cu > W > P > Ce [13]. Choi, W. et al. [14] studied the photocatalytic properties of TiO_2_ doped with 21 transition metals. The results showed that compared with other transition metal ions, Fe^3+^ and Cu ions doping broaden the range of light responded in a certain range, and make the TiO_2_ powder have better catalytic activity. The TiO_2_ doped with iron ions largely determines its optical properties [15]. TiO_2_ doped with Fe^3+^ ions showed better light absorption in the UV and visible regions due to the reduction in band gap energy compared to pure TiO_2_ [16]. The presence of Fe^3+^ doping in TiO_2_ crystal lattice causes the shifted absorption edge towards longer wavelengths where the maximum of solar energy light is located to enhanced response to the visible-light region [10]. In order to study the purification effect of photocatalytic material TiO_2_ on automobile exhaust gas, Kuang, D.L. [17] et al. mixed different amounts of Fe^3+^into TiO_2_ and calcined at 500°C, and then X-ray diffraction measurement and infrared spectrum analysis were carried out. The results showed that the purification efficiency of modified TiO_2_ was higher than that of undoped TiO_2_. For example, the purification rate of CO and hydrocarbon increased by 0.6% and 2.3% respectively. This is because Fe^3+^ can capture electrons and holes at the same time, which reduces the recombination probability of electron-hole pairs.

In addition to experiments, molecular dynamics (MD) simulation is also introduced in the study of photocatalytic materials absorbing automobile exhaust, because it is the computer simulation of a real molecular system, which can obtain the data that is difficult or impossible to obtain in experiments. Based on mechanical theory and computing technology, MD simulation can simulate and predict the composition, structure, inherent properties and performance of materials. Many scholars have used MD to simulate the physical and chemical properties of TiO_2_ and obtain the process information. Predota, M. et al. [18,19] studied the adsorption of water molecules on rutile TiO_2_ (110) surface by the MD method. Koparde, V. N. and Cummings, P. T. [20] also used the MD simulation to study the adsorption of water molecules on rutile and anatase titanium dioxide nanoparticles. Lin, H. F. et al. [21] studied the adsorption of CO on TiO_2_ particles with different crystal forms (rutile and anatase). The simulation result showed that at different temperatures, CO showed stronger adsorption on anatase.

## 2. Preparation of modified TiO_2_ powder

**Fig 1:**
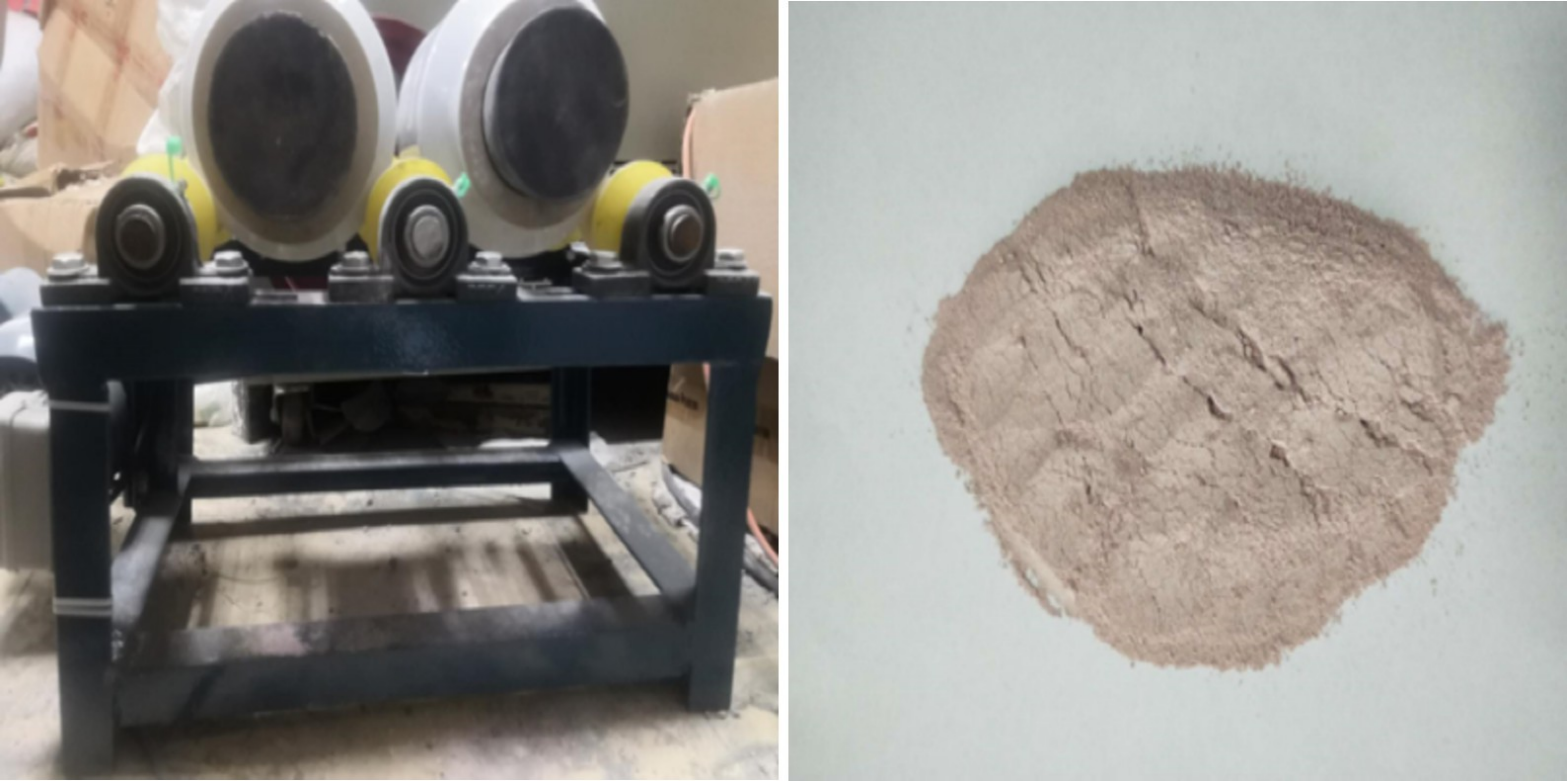
Ball mill and modified TiO_2_ powder.

10g Fe_2_O_3_(99.5% purity) powder and 990g TiO_2_(99.8% purity) powder (the mass ratio is 1:99) were weighed on a precision electronic balance, and the mixed powder was poured into the agate pot of the ball mill. At the same time, some ball-milled balls with a diameter of 1cm were added as the ball milling medium. The mass ratio of the ball-milled beads and the target powder was about 1:1. The rotational speed was set at 300r/min, and the ball milling lasted for about 20h. And then the mixed powder was taken out from the ball mill and put into muffle furnace. Since the nano-TiO_2_ is amorphous without photocatalytic activity, it needs to be heat treated to obtain polycrystalline TiO_2_ in order to improve its catalytic activity [22]. From the research of Abdullah, SA, et al. about nano-TiO_2_ after annealing at different temperatures, it indicates that there were more crystalline phase (mainly including anatase phase and rutile phase) and the proportion of crystalline phases was optimal at 500°C, which can lead to the increase in catalytic activity [23]. In addition to temperature, calcination time is also important. If the time is too long, the structure of titanium dioxide gradually changes from anatase to rutile phase with low photocatalytic activity and the particle size increases. However, if the time is too short, less crystallization phase is not conducive to catalysis. Therefore, according to previous studies and preliminary tests, the heat-treated methods of nano-TiO_2_ is as follow. The temperature was turned to 500°C at the speed of 8°C/min, lasting the temperature for 4h. After that, the target powder was cooled to room temperature and taken out for use (as shown in Fig 1) [22].

The ball milling method for preparing modified TiO_2_ can make iron oxide and titanium dioxide interact to achieve the purpose of ion doping. In the process of ball milling, there is a strong collision between TiO_2_ powders, and plastic deformation occurs, resulting in large stress and strain, which makes the lattice of TiO_2_ seriously distorted. At the same time, a lot of heat is released during the ball milling process, and the strong mechanical force can make Fe^3+^ separated from Fe_2_O_3_ and diffuse into the distorted lattice of TiO_2_. In addition, ball milling can reduce the particle size of TiO_2_, to obtain iron modified nano TiO_2_ powder with a large specific surface area. The method has the advantages of low threshold, simple process and large-scale continuous production.

## 3.Test study

**Fig 2:**
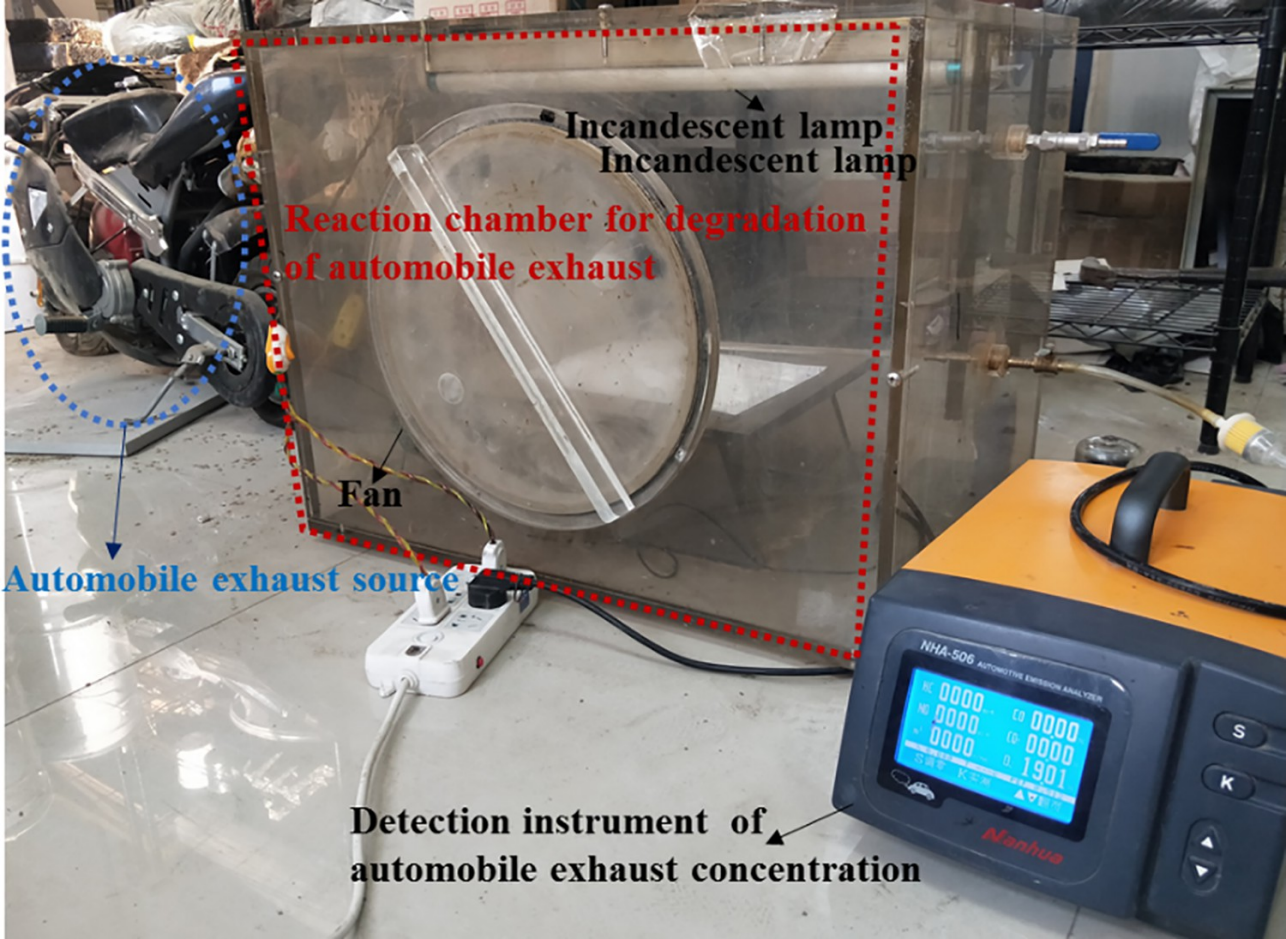
The test device of automobile exhaust.

The test process was as follows: firstly, the room temperature was adjusted to the initial test temperature through air conditioning, then the fan was turned on to prevent gas stratification, and the cover plate was closed. The sealing ring between the cover plate and the tail gas reaction chamber was used to ensure the tightness of the equipment. Secondly, the switch of exhaust gas supply source was turned on to introduce a certain amount of automobile exhaust into the reaction chamber about 10 min. When the vehicle exhaust reached the initial concentration, the switch of exhaust gas was turned off and then the incandescent light was turned up to start the degradation test. The test device is shown in Fig 2. The exhaust gas concentration in the test process was read and recorded by the automobile exhaust gas analyzer every 10 minutes. The test lasted for 60 minutes, so as to analyze the variation law of automobile exhaust gas concentration with time.

## 4. Test method and molecular dynamics simulation method of absorbing automobile exhaust gas

**Fig 3:**
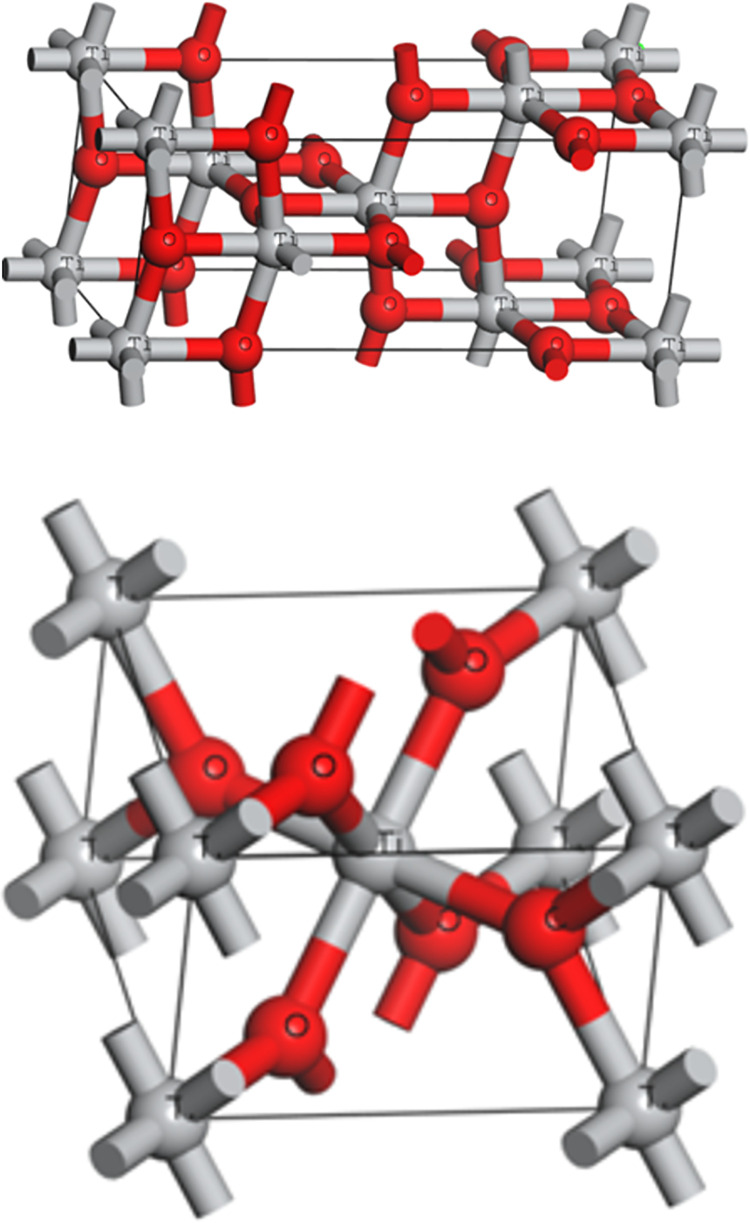
Cell model of TiO_2_.

In this study, the commercial software Materials Studio was used to establish the TiO_2_ pore model to simulate the adsorption of automobile exhaust (CO, NO_2_ and NO). There are three crystal structures of TiO_2_ in nature: brookite, anatase and rutile [24]. Among them, the thermal stability of TiO_2_ with brookite structure and anatase structure is not good, so rutile type TiO_2_ is mainly used in industry. In the XRD spectra made by other scholars of Fe^3+^ modified nano titanium dioxide, the structure of unmodified titanium dioxide is amorphous, and gradually changes to a crystalline structure (anatase and rutile) with the doping of iron ions [16]. Table 1 and Fig 3 show Lattice constants and Cell model of TiO_2_.

**Table 1:** Lattice constants of TiO_2_.

Name	Lattice constants						
	Crossing angle (°)			Volume	Edge length (Å)		
	α	β	γ	Cell Volume	a	b	c
TiO_2__anatase	90	90	90	135.259	3.776	3.776	9.486
TiO_2__rutile	90	90	90	62.4492	4.594	4.594	2.959

In crystallography, Miller indexes are commonly used to determine the cutting index of a single cell by determining three edges as the coordinate system. Taking the lattice side length as the unit, the intercept of a plane on the three axes of the cell rectangular coordinate system is measured, which is expressed as Miller index (h k l). The cutting index of the cell determines the bare atoms on the surface, which directly affects the surface properties of the crystal.

**Fig 4:**
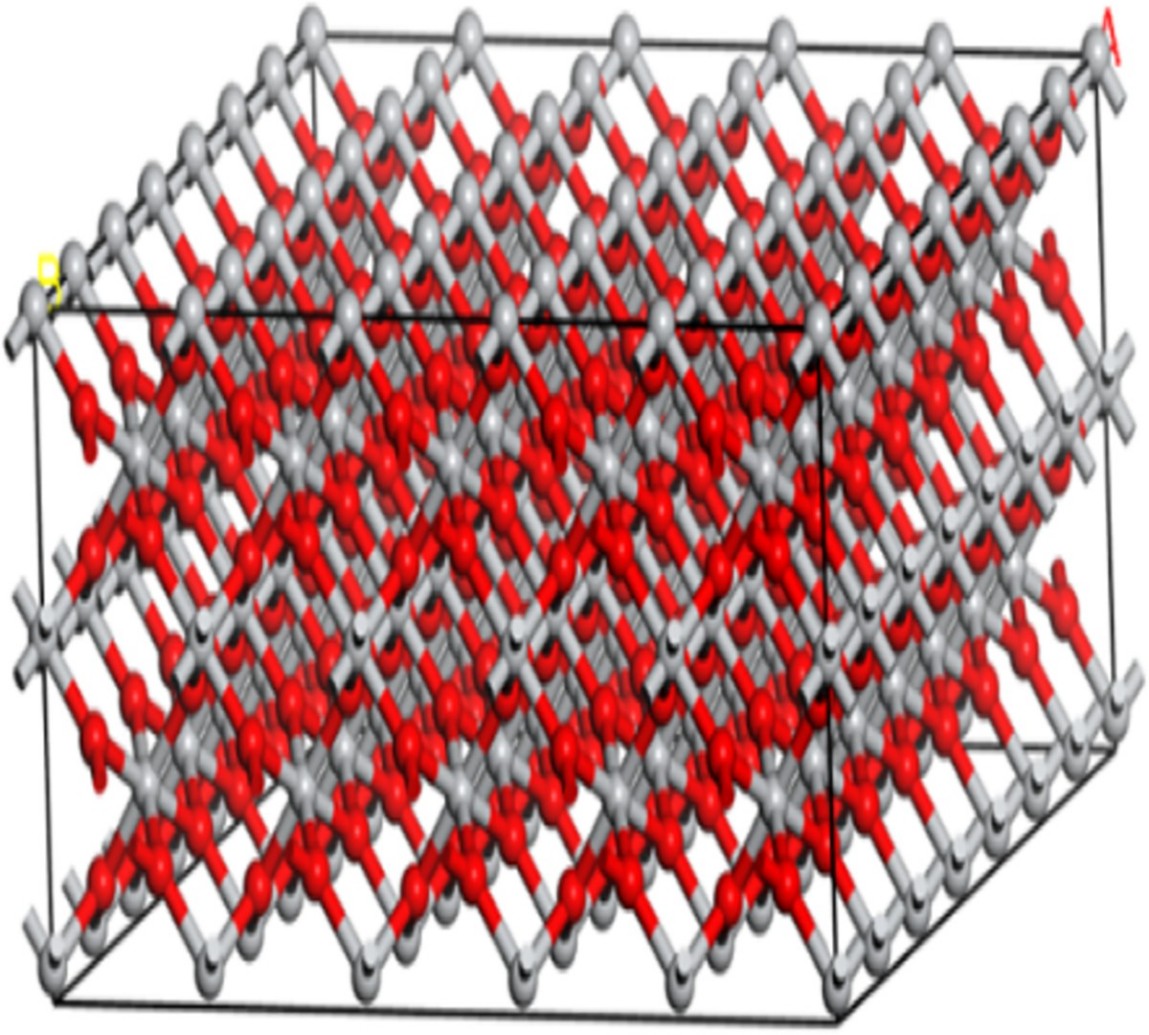
The supercell model of TiO_2_.

In nature, the main Miller indexes of TiO_2_ are (1 0 0) (0 1 0) (0 0 1). From previous studies, the crystal face index (1 0 0) of TiO_2_ is most extensive. Therefore, this paper used the crystal face index to cut TiO_2_. When cutting, the thickness was set to 10 Å and the model was transformed from 2D to 3D by adding a vacuum layer. Then, the supercell tool in the build module of materials studio was used to establish the TiO_2_ supercell model (Fig 4) with a ×b ×C = 5 ×7 ×1 repetitions, so as to expand the original cell of TiO_2_. Finally, the size of the supercell was about 55 Å× 53 Å× 10 Å to realize the contact with automobile exhaust in a larger area and to obtain more reasonable simulation results. According to the composition and mass ratio of each component in automobile exhaust, at the same time, considering the feasibility of molecular dynamics simulation, the number ratio of main components of automobile exhaust was determined as CO: NO: NO_2_: SO_2_: C_20_H_12_ = 90:4:8:2:2.

**Fig 5:**
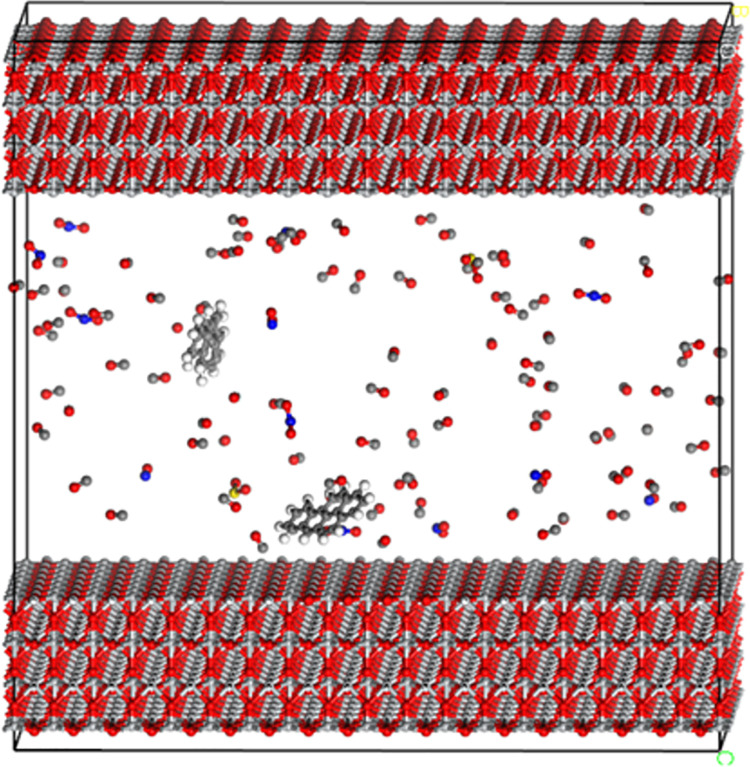
The pore model of TiO_2_ absorbing automobile exhaust.

TiO_2_ powder usually has some microporous structure, which is conducive to adsorption of automobile exhaust. Based on this, through the build layer tool in Materials Studio software, this paper built the pore model unit of TiO_2_ absorbing automobile exhaust gas by superposition in the order of TiO_2_, automobile exhaust and TiO_2_, as shown in Fig 5. Compared with unmodified TiO_2_, Fe^3+^ ion modified TiO_2_ is different in that the trivalent Fe replaces the tetravalent Ti atom, which leads to the imbalance of charge and shows strong external activity.

The setting of several important parameters in the simulation process: inorganic TiO_2_ and organic gases were both included in the model, so, compass force field which can not only calculate metal inorganic compounds but also analyze organic compounds was adopted in this study. In the model, because the number of particles remained unchanged and the pore volume remained constant, the constant volume and temperature (NVT) ensemble was selected to simulate the absorption of vehicle exhaust gas by TiO_2_ at a certain temperature. After the size of a single box was determined, a reasonable cut-off radius which refers to the effective interaction distance between atoms should be set in the simulation process. Generally, the cut-off radius should not be greater than half of the box size to avoid repeated calculation [25,26]. So, in this paper, the cut-off radius was set as 15.5 Å, which can avoid repeated calculation and achieve good simulation accuracy.

In order to get the equilibrium of the dynamic simulation quickly, the energy optimization of the channel model was carried out and the model was preliminarily optimized. At the same time, before MD simulation, the upper and lower TiO_2_ molecular layers were fixed, and the pore model was fully relaxed to simulate the adsorption of TiO_2_ on automobile exhaust, which made the simulation closer to the real situation. The NVT ensemble and compass II force field were used to calculate the MD equilibrium of 100 PS at a standard atmospheric pressure at 298K and 333K respectively, and the simulation results at ambient temperature and high temperature were obtained respectively.

**Fig 6:**
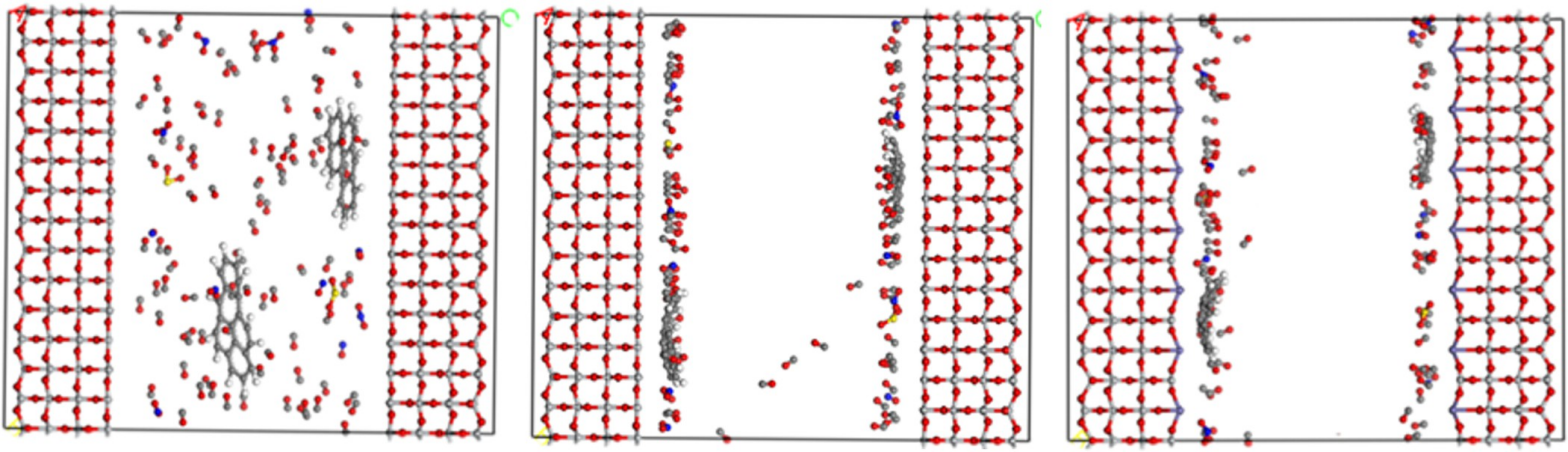
Adsorption on exhaust molecules of unmodified and modified TiO_2_ on automobile exhaust before and after MD equilibrium in TiO_2_ channel. (**a**) Unmodified TiO_2_ before MD (**b**) Unmodified TiO_2_ before MD (**c**) Modified TiO_2_ after MD.

After molecular dynamics calculation, the equilibrium adsorption model of automobile exhaust gas by titanium dioxide was obtained. Fig 6 shows the comparison of adsorption effects of unmodified TiO_2_ and Fe^3+^ ion modified TiO_2_ on automobile exhaust gas from the perspective of visualization after MD equilibrium. It can be seen that most of the automobile exhaust molecules orderly approach to the surface of TiO_2_. The CO molecule changed from a disordered state to "standing" on the surface of TiO_2_, and the C atom end was close to TiO_2_, and the O atom end was far away from TiO_2_. The main reason is that there are 10 valence electrons in the CO molecule, which form three chemical bonds: one σ bond and two π bond. One of the two π bonds is a coordination bond, and the bonding electrons are completely provided by O atoms. C atoms in CO molecules have a slightly negative charge, which makes the arc electron pairs on C atoms easily enter the empty orbits of other atoms and form coordination bonds.

Therefore, in the reaction, CO is an electron pair donor and can form complexes with some atoms with empty orbitals. However, for NO and NO_2_, O atoms are closer to the surface of TiO_2_, and N atoms are slightly separated. There is no obvious regularity in the distribution of SO_2_ in the pore.

## 5. Results and discussion

### 5.1. Analysis of test result

In order to effectively analyze the degradation effect of powder on automobile exhaust, the degradation efficiency was used as the evaluation index. and the degradation ratio of exhaust gas was evaluated in different periods. The calculation formula is shown in Eq (1) [27], and the calculation results are shown in Table 2.

$$D=\frac{C_{before}-C_{after}}{C_{before}}\times 100\%$$ (1)

Where *D* is the degradation ratio of gas in the target stage, *C*_*before*_ is the initial concentration, *C*_*after*_ is the end concentration.

**Table 2:** Concentration variation and degradation ratio of vehicle exhaust gas.

Types of gas		Test time (min)							Variation	The degradation efficiency (%)
		0	10	20	30	40	50	60		
NO (ppm)	Blank group	57	56	58	59	57	56	56	-1	1.75%
	TiO_2_	54	53	40	38	36	33	32	-22	40.74%
	Modified TiO_2_	52	46	35	29	22	10	4	-48	92.31%
NO_2_ (ppm)	Blank group	41	42	42	41	42	41	40	-1	2.44%
	TiO_2_	46	48	53	54	50	46	41	-5	10.87%
	Modified TiO_2_	43	46	51	53	48	41	38	-5	11.63%
C_x_H_y_	Blank group	275	274	277	274	272	272	274	-1	0.36%
	TiO_2_	282	282	276	268	260	258	254	-28	9.93%
	Modified TiO_2_	278	278	262	258	254	252	250	-28	10.07%
CO (%)	Blank group	4.69	4.75	4.72	4.66	4.67	4.69	4.67	-0.02	0.43%
	TiO_2_	4.56	4.52	4.44	4.38	4.24	4.22	4.2	-0.36	7.89%
	Modified TiO_2_	4.72	4.55	4.38	4.28	4.12	4.06	3.87	-0.85	18.01%
CO_2_ (%)	Blank group	4.56	4.54	4.57	4.58	4.6	4.57	4.55	-0.01	0.22%
	TiO_2_	4.42	4.41	4.65	4.73	4.52	4.43	4.2	-0.22	4.98%
	Modified TiO_2_	4.72	4.69	4.77	4.82	4.74	4.34	4.26	-0.46	9.75%

**Fig 7:**
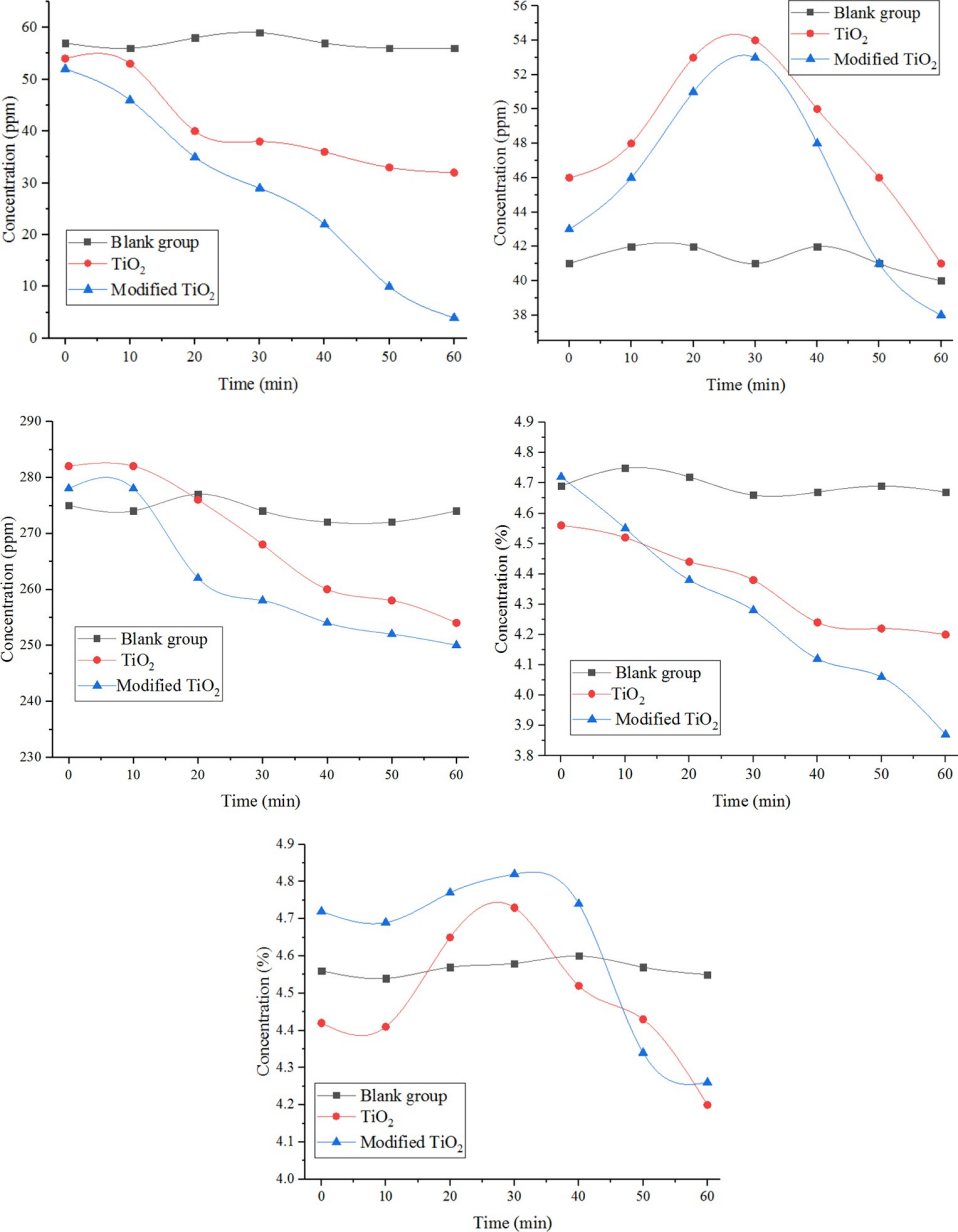
Variation curve of concentration of automobile exhaust. (a) Concentration variation of NO during degradation process. (b) Concentration variation of NO_2_ during degradation process. (c) Concentration variation of C_x_H_y_ during degradation process. (d) Concentration variation of CO during degradation process. (e) Concentration variation of CO_2_ during degradation process.

It can be seen from Table 2 and Fig 7 that, compared with the blank group, ordinary TiO_2_ powder has some degradation effect on CO, NO, NO_2_ and C_x_H_y_ in automobile exhaust. After 60 min, the degradation efficiency of these gas reached 7.89%, 40.74%, 10.87% and 9.93%, respectively. It can be seen that TiO_2_ powder had good degradation effect on NO. At the same time, a part of NO_2_, C_x_H_y_ and CO was also degraded. After TiO_2_ modified by Fe^3+^ions, the degradation effect of NO was significantly improved (increase by 51.57%). Due to its extrinsic bandgap with low energy, Fe^3+^ incorporated into the crystal structure of TiO_2_ promotes electron-hole separation, and deposits active sites on the surface of TiO_2_, thus improving the adsorption of small molecular weight of NO with fast diffusion velocity [28]. Also, there is some positive effect about the degradation of CO (increase by 10.11%). Yet, there is no significant improvement in the degradation of other gases, such as NO_2_, C_x_H_y_. During the process of automobile exhaust degradation, the concentration of NO_2_ and CO_2_ increases first and then decreased. The main reason is that, in the first half, the modified TiO_2_ powder oxidizes harmful gas NO and CO into NO_2_ and non-toxic CO_2_, and the amount of NO_2_ andCO_2_ generated by oxidation may be more than that of purified NO_2_ and CO_2_, so the concentration is going up slightly [29–31]. In the latter half, the NO_2_ and CO_2_ are photocatalyzed into organic matter or salts, thus reducing the concentration.

### 5.2. Simulation analysis of automobile exhaust diffusion in the pore of TiO_2_

Diffusion analysis is a statistical analysis of the migration of materials in space caused by the thermal movement of a large number of molecules and atoms. The molecular diffusion coefficient is often used to characterize the diffusion velocity. The molecular diffusion coefficient which is often used to characterize the diffusion rate is the proportional constant of the concentration difference between two points and the distance, when a component molecule is transferred from a high concentration place to a low concentration place. For a long time, one-sixth of the slope of the MSD curve is the diffusion coefficient of the system. Therefore, in MS software, the MSD is used to analyze the diffusion velocity of the system, and the calculation formula is Eq (2) [32–35].

$$MSD=<{\left|r(t)‐r(0)\right|}^{2}>$$ (2)

Where *r(t)* is the displacement of particles at time t (m); *r(0)* is the displacement of particles at the initial time (m); *< >* is the average of all atoms in the system.

**Fig 8:**
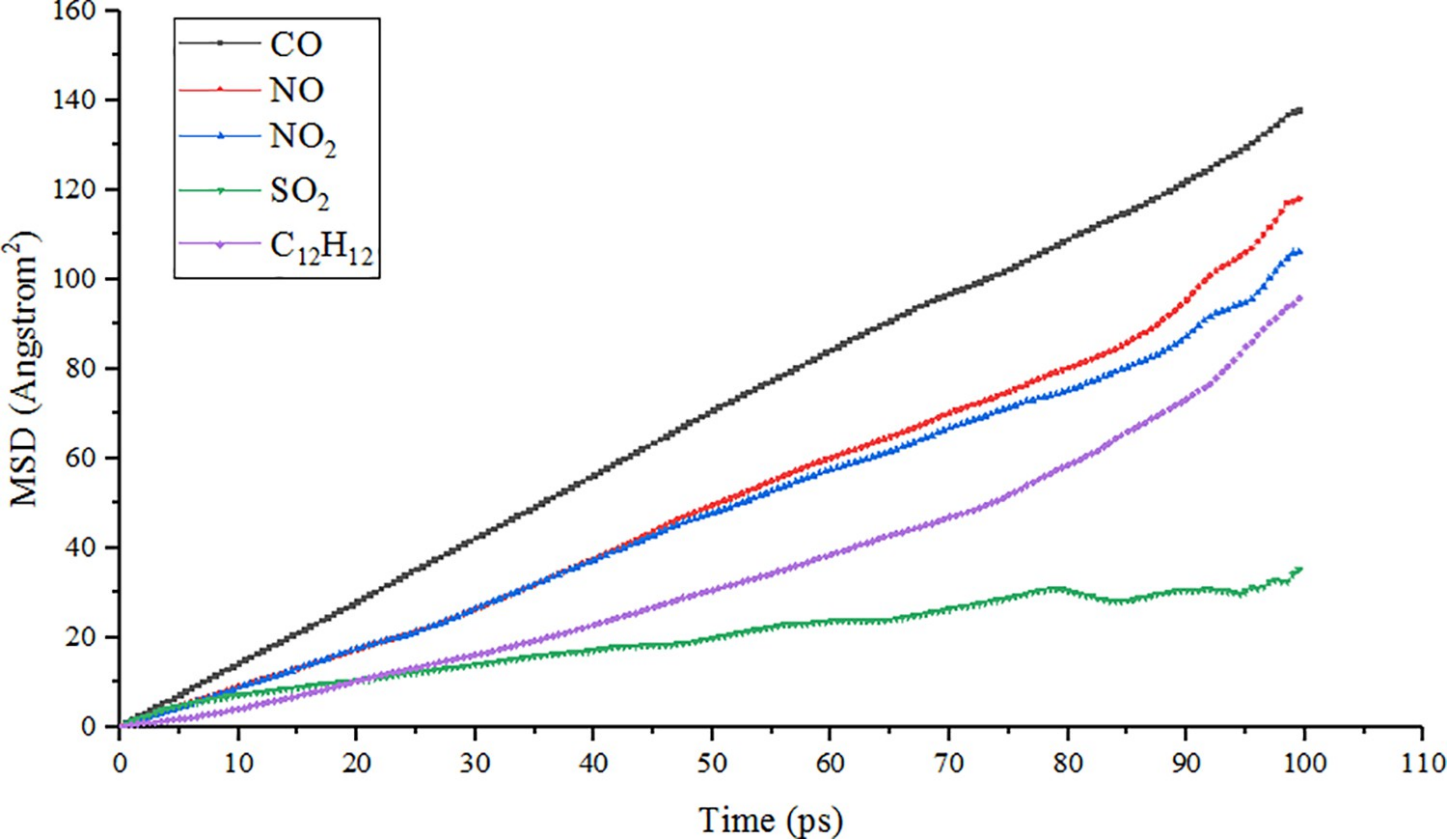
Diffusion velocity of automobile exhaust gas in modified TiO_2_ channel at ambient temperature (298K).

**Fig 9:**
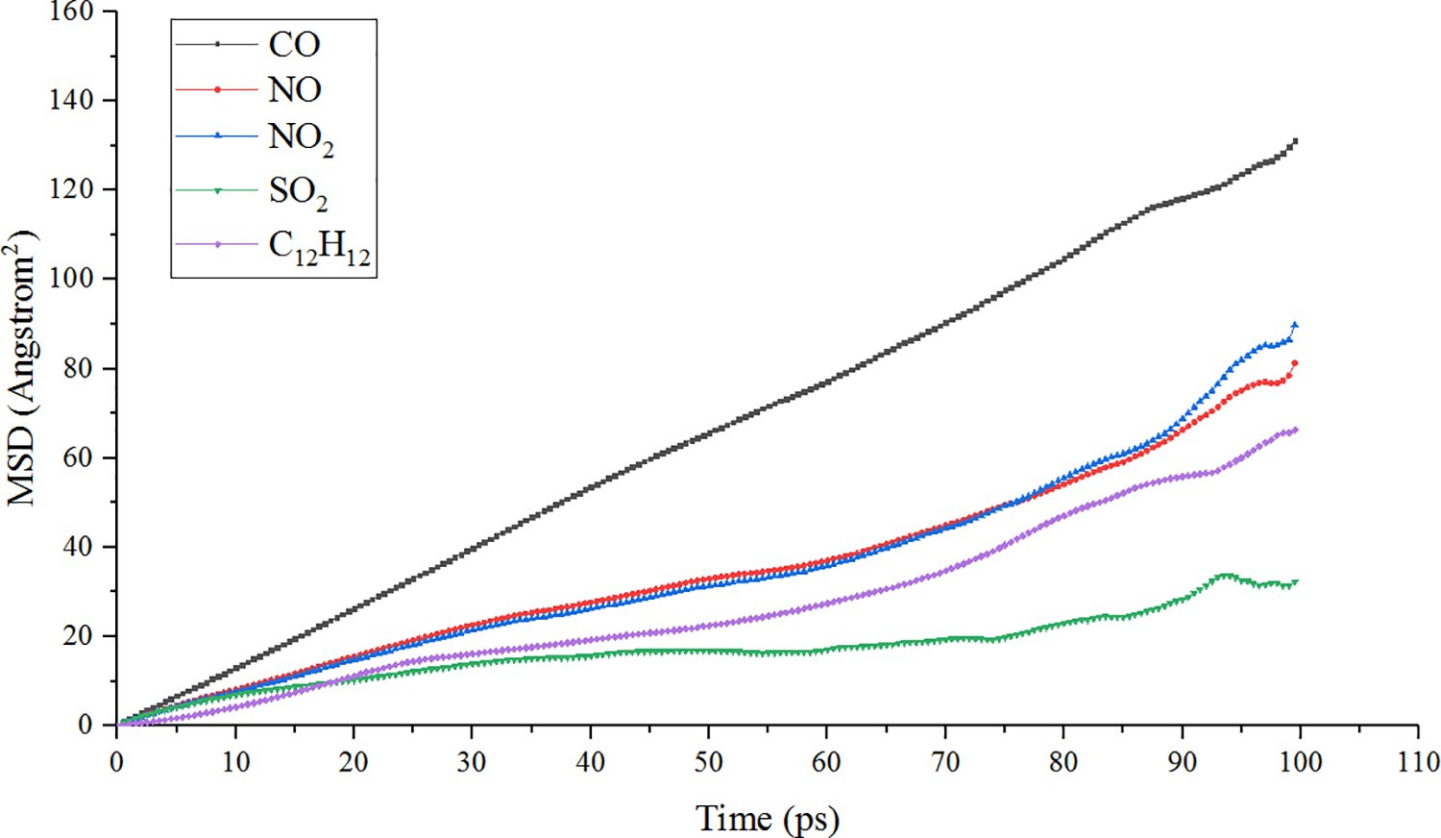
Diffusion velocity of automobile exhaust gas in unmodified TiO_2_ channel at ambient temperature (298K).

The diffusion velocity of different types of automobile exhaust molecules in the modified and unmodified TiO_2_ channels are shown in Figs 8 and 9, respectively. It can be seen from the figure that the diffusion velocity of CO molecules with the smallest molecular weight was the fastest. The second was NO and NO_2_, and the diffusion rates of these two gases were very close. The third was C_12_H_12_, which had the largest molecular weight because the heavy molecule slowed down the diffusion velocity due to its large volume and weight. The molecule of the gas with the slowest diffusion speed was SO_2_, and this law was consistent in the modified and unmodified TiO_2_ channels. It shows that the adsorption powder of TiO_2_ for SO_2_ molecules was weaker than other exhaust molecules. From the energy point of view, the diffusion process is caused by energy gradient, and it is also the process of system transition from the unsteady state (high energy) to steady-state (low energy). It also reflects the change of various forces when the system tends to be stable.

At the same time, the diffusion velocity of CO, NO, NO_2_ and C_12_H_12_ molecules in the modified TiO_2_ channel was faster than that in the unmodified TiO_2_ channel, while the adsorption of SO_2_ molecules had almost no change. The results show that the modification of TiO_2_ improved its attraction to most automobile exhaust molecules and promoted the directional diffusion of these molecules to the surface of TiO_2_. This is due to the incorporation with Fe^3+^, there are more active sites on the surface of titanium dioxide, which accelerates the adsorption of exhaust gas molecules, thus promoting the diffusion of exhaust gas molecules on the crystal surface [36].

### 5.3. Radial concentration analysis of automobile exhaust on TiO_2_ surface

The concentration distribution of the molecule on the crystal surface is an important index of the adsorption of the molecule by the crystal. The closer the adsorption distance and the higher the concentration, the better the adsorption effect between the molecule and crystal [37]. In MD simulation, the radial distribution function (g(r)), which can be explained as the ratio of local density to average density bulk density of the system, is often used to characterize the cross-sectional concentration distribution of particles.

In this study, the geometric center of the TiO_2_ layer was used as the reference point to calculate the distance between automobile exhaust molecules and TiO_2_. The shorter the distance and the higher the concentration, the better the absorption effect of automobile exhaust, and the less easy to release. The calculation formula of the radial distribution function is Eq (3) [38].

$$g(r)=\frac{1}{\rho 4\pi r^{2}\delta r}\frac{\sum_{t=1}^{T}\sum_{j=1}^{N}\Delta N(r\to r+\delta r)}{N\times T}$$ (3)

where *N* is the total number of molecules (number); *T* is the total calculation time (steps); *δ*_*r*_ is the designed difference in the distance; and *△N* is the number of molecules within the interval of *r→r+δ*_*r*_.

**Fig 10:**
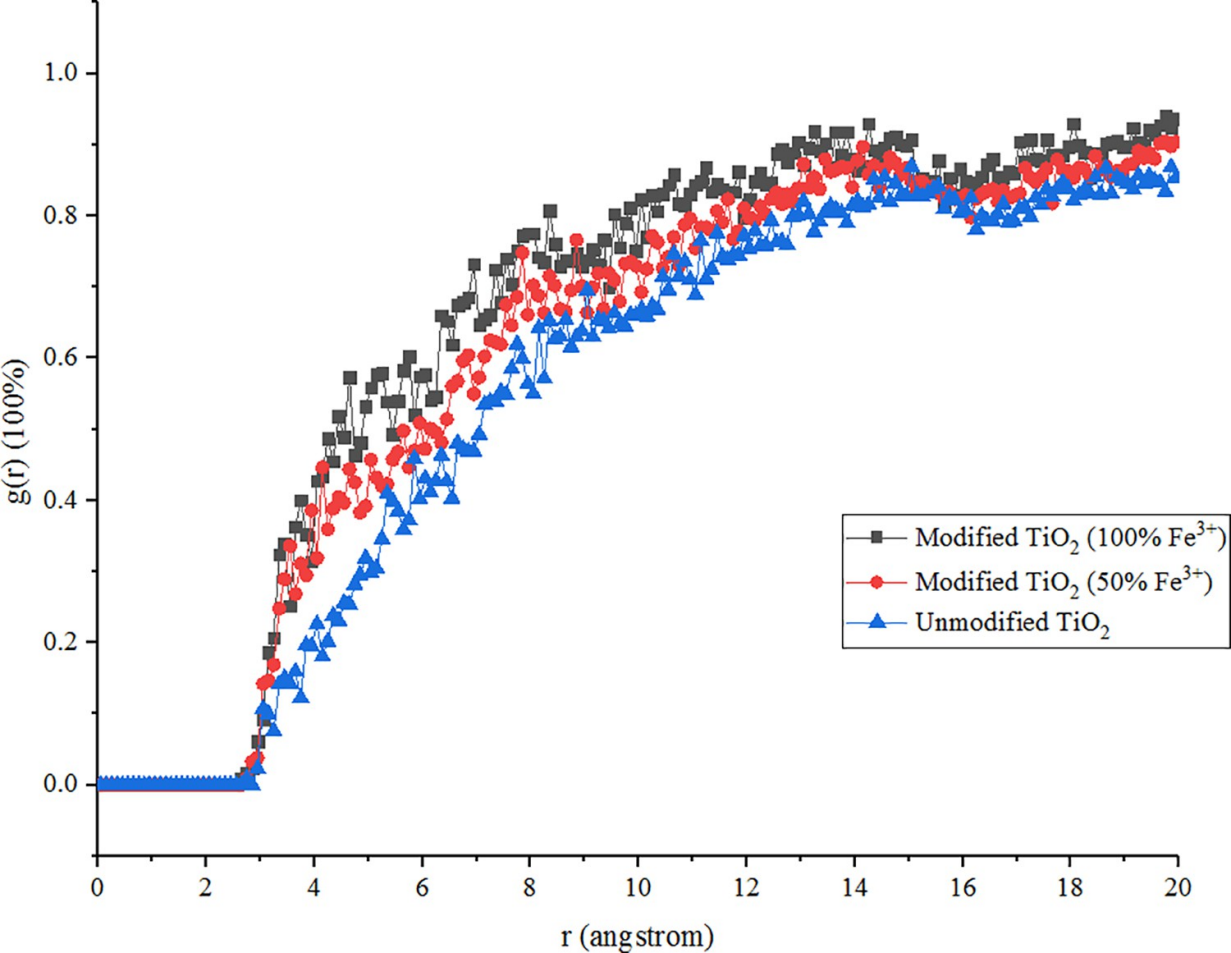
Radial relative concentration of automobile exhaust on different types of TiO_2_ surface.

Fig 10 shows the radial distribution concentration of automobile exhaust on the surface of TiO_2_ powder under three conditions (unmodified TiO_2_, TiO_2_ modified by Fe^3+^ on the surface of 50% Ti atom and TiO_2_ modified by Fe^3+^ on surface of 100% Ti atom). It can be seen that after modification, the radial concentration of automobile exhaust absorption at the same position in the TiO_2_ channel has been significantly increased, and the order of concentration is 100% Fe^3 +^ modified TiO_2_ > 50% Fe^3 +^ modified TiO_2_ > unmodified TiO_2_. Especially, the difference was more obvious in the near end of TiO_2_. This may be attributed to the fact that the titanium dioxide fully modified by iron ions with low bandgap promotes the separation of electron/hole and further improves the catalytic efficiency [39]. This phenomenon shows that Fe^3 +^ can effectively improve the adsorption of TiO_2_ powder to automobile exhaust, and the more complete the modification, the better the effect.

### 5.4. Calculation and analysis of adsorption energy of automobile exhaust on TiO_2_ surface

In the previous section, the improvement effect of modified TiO_2_ on automobile exhaust gas adsorption was analyzed from the perspective of radial relative concentration, and the adsorption strength was determined from the perspective of distance and concentration. This section will quantitatively analyze the absorption effect of modified TiO_2_ on automobile exhaust from the perspective of energy.

When the automobile exhaust gas is adsorbed in the TiO_2_ channel, the two independent systems of vehicle exhaust and TiO_2_ gradually become a whole system, and refer to other systems, the reduction of system energy is considered as the adhesion work [40]. In other words, the adhesion work is the difference between the total energy of a whole-body (formed by adsorption of automobile exhaust and TiO_2_) and the sum of the surface energy of vehicle exhaust and TiO_2_ respectively before adsorption. The larger the adhesion work value between automobile exhaust and TiO_2_, the more difficult the automobile exhaust to separate from the surface of TiO_2_, which means that the adsorption is more difficult to be destroyed [41,42]. The calculation method is shown in Eq (4).

$$W_{adhension}=\frac{\Delta E}{A}\times K=\frac{E_{total}-(E_{TiO_{2}}+E_{gas})}{A}\times K$$ (4)

where *W*_*adhension*_ represents the work of adhesion at the interface (mJ m^-2^). *E*_*total*_ represents the interfacial potential energy of TiO_2_ and automobile exhaust after molecular dynamic adsorption equilibrium (kcal mol^-1^). *E*_*gas*_ is the potential values of the automobile exhaust after MD simulation (kcal mol^-1^). *E*_*TiO2*_ are the potential values of the TiO_2_ after MD simulation (kcal mol^-1^). *A* represents the Connolly area of the interface (Å^2^). *△E* represents the Variation value of automobile exhaust gas during the adsorption process (kcal mol^-1^).K = 695 [43] is the conversion coefficient for units.

**Fig 11:**
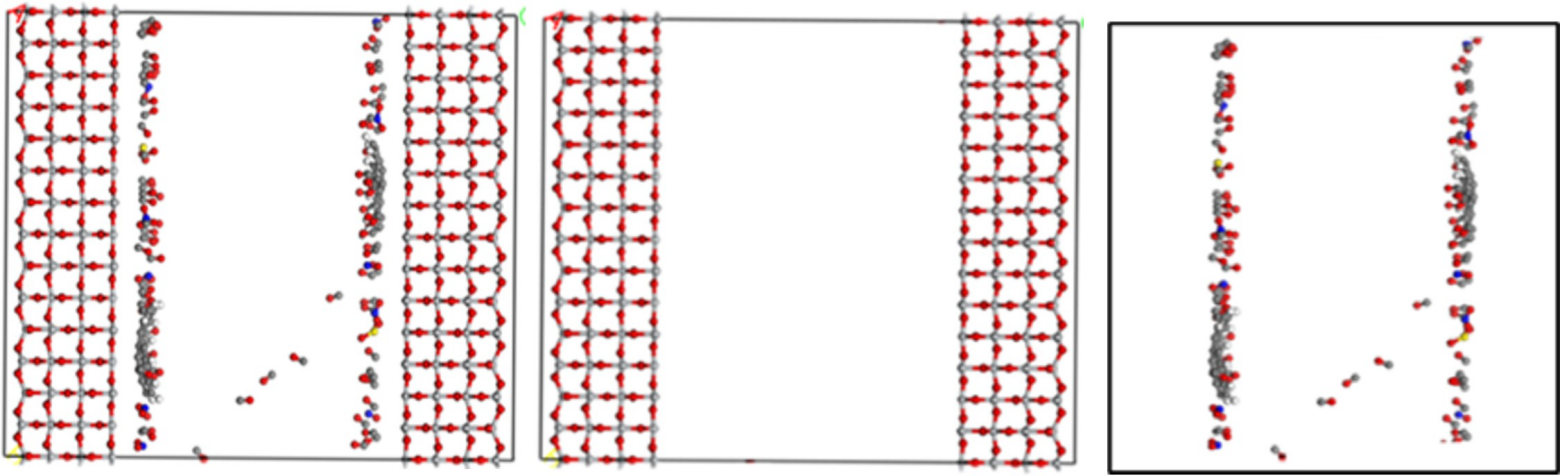
Disassembled model after MD equilibrium. (a) Automobile exhaust in TiO_2_ channel (b) TiO_2_ (c) Automobile exhaust.

After the MD equilibrium calculation of the two-phase interface model of automobile exhaust (gas) and TiO_2_ (solid), the model of TiO_2_ adsorption on automobile exhaust was built. And it is shown in Fig 11(a). The interface model was divided into two parts: TiO_2_ model and automobile exhaust model. The energy function of force module in Materials Studio software was used to calculate the energy of interface model (a), the disassembled TiO_2_ model (b) and automobile exhaust model (c) respectively, so as to calculate the adhesion work between TiO_2_ and automobile exhaust gas using the above formula. The calculation results are shown in Table 3. The results present that the adhesion work between the automobile exhaust and TiO_2_ was obviously improved. The 50% Fe^3+^ and 100% Fe^3+^modified TiO_2_ increased about 40% and 73%, respectively. The incorporation of Fe^3+^ into the structure of TiO_2_, causing the reduction of the bandgap, the prevention of recombination of electron/hole and extension of the life of charge separation, increases the number of active points and absorbs more exhaust molecules, which results in the increase of adhesion work [36]. Therefore, Fe^3+^ modification is beneficial to enhance the adsorption capacity of TiO_2_ on automobile exhaust, thus improving the degradation effect.

**Table 3:** Calculation of adhesion work.

	△E (kcalmol^-1^)	△E_vdw_ (kcalmol^-1^)	△E_ele_ (kcalmol^-1^)	A Å^2^	W_adhension_ (mJ/m^2^)
TiO_2_-Gas	-573.888	-395.64	-104.928	4844.53	82.33
Modified TiO_2_ (50%Fe^3+^)-Gas	-808.366	-251.862	-107.961	4862.24	115.55
Modified TiO_2_ (100%Fe^3+^)-Gas	-1002.358	-371.977	-216.059	4874.12	142.93

## 6. Conclusions

In this study, Fe^3+^ was used to modify the photocatalyst material TiO_2_. Through the absorption test of automobile exhaust and the diffusion and absorption of automobile exhaust gas in the TiO_2_ channel simulated by molecular dynamics method, it is proved that the modification can effectively improve the degradation effect of TiO_2_ on automobile exhaust. The conclusions are as follows:

1. Test results show that the modification of TiO_2_ can obviously improve the degradation effect of NO in automobile exhaust, but it is not obvious to improve the degradation effect of other exhaust gases such as CO and NO_2_.
2. MD simulation shows that the diffusion velocity of most automobile exhaust molecules in the modified TiO_2_ channels is faster than that in the unmodified TiO_2_ channels, which indicates that the modification of TiO_2_ improves the attraction of most automobile exhaust molecules and promotes the directional diffusion of automobile exhaust molecules to the surface of TiO_2_.
3. Through MD simulation and analysis of the radial concentration and the adsorption energy of exhaust molecules in the TiO_2_ channel, it is concluded that Fe^3 +^ modification of TiO_2_ powder is beneficial to improve the adsorption of automobile exhaust molecules, and the more the modification, the better the effect.

## Supporting information

S1 File S1 File contains the original data for Figs 8–10. (XLSX)
